# The Endocrine System: An Overview

**Published:** 1998

**Authors:** Susanne Hiller-Sturmhöfel, Andrzej Bartke

**Affiliations:** Susanne Hiller-Sturmhöfel, Ph.D., is a science editor of Alcohol Health & Research World. Andrzej Bartke, Ph.D., is professor and chairman of physiology at Southern Illinois University School of Medicine, Carbondale, Illinois

**Keywords:** endocrine function, hormones, hypothalamus, pituitary gland, gonad function, thyroid, parathyroid, pancreas, biochemical mechanism, biological feedback, biological regulation, hypothalamus-pituitary axis, pituitary-adrenal axis, pituitary-thyroid axis, literature review

## Abstract

A plethora of hormones regulate many of the body’s functions, including growth and development, metabolism, electrolyte balances, and reproduction. Numerous glands throughout the body produce hormones. The hypothalamus produces several releasing and inhibiting hormones that act on the pituitary gland, stimulating the release of pituitary hormones. Of the pituitary hormones, several act on other glands located in various regions of the body, whereas other pituitary hormones directly affect their target organs. Other hormone-producing glands throughout the body include the adrenal glands, which primarily produce cortisol; the gonads (i.e., ovaries and testes), which produce sex hormones; the thyroid, which produces thyroid hormone; the parathyroid, which produces parathyroid hormone; and the pancreas, which produces insulin and glucagon. Many of these hormones are part of regulatory hormonal cascades involving a hypothalamic hormone, one or more pituitary hormones, and one or more target gland hormones.

For the body to function properly, its various parts and organs must communicate with each other to ensure that a constant internal environment (i.e., homeostasis) is maintained. For example, neither the body temperature nor the levels of salts and minerals (i.e., electrolytes) in the blood must fluctuate beyond preset limits. Communication among various regions of the body also is essential for enabling the organism to respond appropriately to any changes in the internal and external environments. Two systems help ensure communication: the nervous system and the hormonal (i.e., neuroendocrine) system. The nervous system generally allows rapid transmission (i.e., within fractions of seconds) of information between different body regions. Conversely, hormonal communication, which relies on the production and release of hormones from various glands and on the transport of those hormones via the bloodstream, is better suited for situations that require more widespread and longer lasting regulatory actions. Thus, the two communication systems complement each other. In addition, both systems interact: Stimuli from the nervous system can influence the release of certain hormones and vice versa.

Generally speaking, hormones control the growth, development, and metabolism of the body; the electrolyte composition of bodily fluids; and reproduction. This article provides an overview of the hormone systems involved in those regulatory processes. The article first summarizes some of the basic characteristics of hormone-mediated communication within the body, then reviews the various glands involved in those processes and the major hormones they produce. For more in-depth information on those hormones, the reader should consult endocrinology textbooks (e.g., Constanti et al. 1998; Wilson et al. 1998). Finally, the article presents various endocrine systems in which hormones produced in several organs cooperate to achieve the desired regulatory effects. The discussions focus primarily on the system responses in normal, healthy people. For information regarding alcohol’s effects on some of the hormone systems, the reader is referred to subsequent articles in this issue of *Alcohol Health & Research World*.

## What Are Hormones?

**Figure 1:**
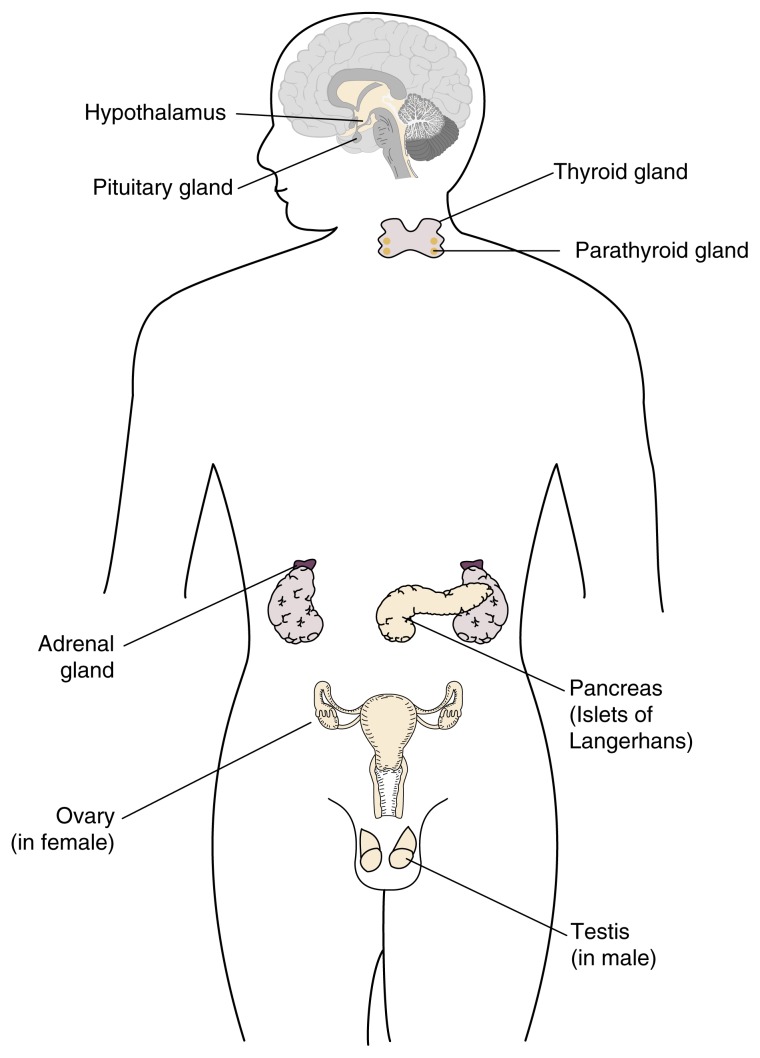
Schematic representation of the location of the major hormone-producing (i.e., endocrine) organs in the body. (For the purposes of illustration, both male and female endocrine organs are presented here.)

Hormones are molecules that are produced by endocrine glands, including the hypothalamus, pituitary gland, adrenal glands, gonads, (i.e., testes and ovaries), thyroid gland, parathyroid glands, and pancreas (see figure 1). The term “endocrine” implies that in response to specific stimuli, the products of those glands are released into the bloodstream.^1^ The hormones then are carried via the blood to their target cells. Some hormones have only a few specific target cells, whereas other hormones affect numerous cell types throughout the body. The target cells for each hormone are characterized by the presence of certain docking molecules (i.e., receptors) for the hormone that are located either on the cell surface or inside the cell. The interaction between the hormone and its receptor triggers a cascade of biochemical reactions in the target cell that eventually modify the cell’s function or activity.

### Mechanisms of Action

Several classes of hormones exist, including steroids, amino acid derivatives, and polypeptides and proteins. Those hormone classes differ in their general molecular structures (e.g., size and chemical properties). As a result of the structural differences, their mechanisms of action (e.g., whether they can enter their target cells and how they modulate the activity of those cells) also differ. Steroids, which are produced by the gonads and part of the adrenal gland (i.e., the adrenal cortex), have a molecular structure similar to that of cholesterol. The molecules can enter their target cells and interact with receptors in the fluid that fills the cell (i.e., the cytoplasm) or in the cell nucleus. The hormone-receptor complexes then bind to certain regions of the cell’s genetic material (i.e., the DNA), thereby regulating the activity of specific hormone-responsive genes.

Amino acid derivatives are modified versions of some of the building blocks of proteins. The thyroid gland and another region of the adrenal glands (i.e., the adrenal medulla) produce this type of hormone (i.e., the amino acid derivatives). Like steroids, amino acid derivatives can enter the cell, where they interact with receptor proteins that are already associated with specific DNA regions. The interaction modifies the activity of the affected genes.

Polypeptide and protein hormones are chains of amino acids of various lengths (from three to several hundred amino acids). These hormones are found primarily in the hypothalamus, pituitary gland, and pancreas. In some instances, they are derived from inactive precursors, or pro-hormones, which can be cleaved into one or more active hormones. Because of their chemical structure, the polypeptide and protein hormones cannot enter cells. Instead, they interact with receptors on the cell surface. The interaction initiates biochemical changes in either the cell’s membrane or interior, eventually modifying the cell’s activity or function.

### Regulation of Hormone Activity

**Figure 2:**
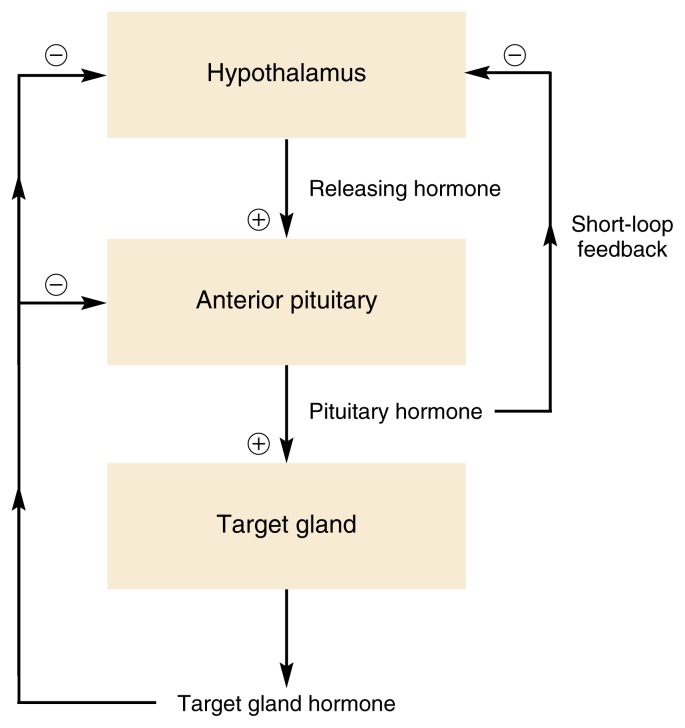
Schematic representation of negative feedback mechanisms that control endocrine system activity. In many cases, the hormones released from the target gland act back on the pituitary and/or hypothalamus, repressing further hormone release from both organs and thereby shutting off the system. For a short-loop negative feedback mechanism, pituitary hormones act directly back on the hypothalamus, inhibiting the release of hypothalamic hormones. NOTE: ⊕ = stimulates; ⊖ = inhibits.

To maintain the body’s homeostasis and respond appropriately to changes in the environment, hormone production and secretion must be tightly controlled. To achieve this control, many bodily functions are regulated not by a single hormone but by several hormones that regulate each other (see figure 2). For example, for many hormone systems, the hypothalamus secretes so-called releasing hormones, which are transported via the blood to the pituitary gland. There, the releasing hormones induce the production and secretion of pituitary hormones, which in turn are transported by the blood to their target glands (e.g., the adrenal glands, gonads, or thyroid). In those glands, the interaction of the pituitary hormones with their respective target cells results in the release of the hormones that ultimately influence the organs targeted by the hormone cascade.

Constant feedback from the target glands to the hypothalamus and pituitary gland ensures that the activity of the hormone system involved remains within appropriate boundaries. Thus, in most cases, negative feedback mechanisms exist by which hormones released by the target glands affect the pituitary gland and/or hypothalamus (see figure 2). When certain predetermined blood levels of those hormones are reached, the hypothalamus and/or the pituitary ceases hormone release, thereby turning off the cascade. In some instances, a so-called short-loop feedback occurs, in which pituitary hormones directly act back on the hypothalamus.

The sensitivity with which these negative feedback systems operate (i.e., the target hormone levels that are required to turn off hypothalamic or pituitary hormone release) can change at different physiological states or stages of life. For example, the progressive reduction in sensitivity of the hypothalamus and pituitary to negative feedback by gonadal steroid hormones plays an important role in sexual maturation.

Although negative feedback is more common, some hormone systems are controlled by positive feedback mechanisms, in which a target gland hormone acts back on the hypothalamus and/or pituitary to increase the release of hormones that stimulate the secretion of the target gland hormone. One such mechanism occurs during a woman’s menstrual period: Increasing estrogen levels in the blood temporarily stimulate, rather than inhibit, hormone release from the pituitary and hypothalamus, thereby further increasing estrogen levels and eventually leading to ovulation. Such a mechanism requires a specific threshold level, however, at which the positive feedback loop is turned off in order to maintain a stable system.

**Table:** Hormones Produced by the Major Hormone-Producing (i.e., Endocrine) Glands and Their Primary Functions

Endocrine Gland	Hormone	Primary Hormone Function
Hypothalamus	Corticotropin-releasing hormone (CRH)	Stimulates the pituitary to release adrenocorticotropic hormone (ACTH)
	Gonadotropin-releasing hormone (GnRH)	Stimulates the pituitary to release luteinizing hormone (LH) and follicle-stimulating hormone (FSH)
	Thyrotropin-releasing hormone (TRH)	Stimulates the pituitary to release thyroid-stimulating hormone (TSH)
	Growth hormone-releasing hormone (GHRH)	Stimulates the release of growth hormone (GH) from the pituitary
	Somatostatin	Inhibits the release of GH from the pituitary
	Dopamine	Inhibits the release of prolactin from the pituitary

Anterior pituitary gland	ACTH	Stimulates the release of hormones from the adrenal cortex
	LH	In women, stimulates the production of sex hormones (i.e., estrogens) in the ovaries as well as during ovulation; in men, stimulates testosterone production in the testes
	FSH	In women, stimulates follicle development; in men, stimulates sperm production
	TSH	Stimulates the release of thyroid hormone
	GH	Promotes the body’s growth and development
	Prolactin	Controls milk production (i.e., lactation)

Posterior pituitary gland^1^	Vasopressin	Helps control the body’s water and electrolyte levels
	Oxytocin	Promotes uterine contraction during labor and activates milk ejection in nursing women

Adrenal cortex	Cortisol	Helps control carbohydrate, protein, and lipid metabolism; protects against stress
	Aldosterone	Helps control the body’s water and electrolyte regulation

Testes	Testosterone	Stimulates development of the male reproductive organs, sperm production, and protein anabolism

Ovaries	Estrogen (produced by the follicle)	Stimulates development of the female reproductive organs
	Progesterone (produced by the corpus luteum)	Prepares uterus for pregnancy and mammary glands for lactation

Thyroid gland	Thyroid hormone (i.e., thyroxine [T_4_] and triiodothyronine [T_3_])	Controls metabolic processes in all cells
	Calcitonin	Helps control calcium metabolism (i.e., lowers calcium levels in the blood)

Parathyroid gland	Parathyroid hormone (PTH)	Helps control calcium metabolism (i.e., increases calcium levels in the blood)

Pancreas	Insulin	Helps control carbohydrate metabolism (i.e., lowers blood sugar levels)
	Glucagon	Helps control carbohydrate metabolism (i.e., increases blood sugar levels)

1 These hormones are produced in the hypothalamus but stored in and released from the posterior pituitary gland.

## The Hypothalamus and Its Hormones

The hypothalamus is a small region located within the brain that controls many bodily functions, including eating and drinking, sexual functions and behaviors, blood pressure and heart rate, body temperature maintenance, the sleep-wake cycle, and emotional states (e.g., fear, pain, anger, and pleasure). Hypothalamic hormones play pivotal roles in the regulation of many of those functions.

Because the hypothalamus is part of the central nervous system, the hypothalamic hormones actually are produced by nerve cells (i.e., neurons). In addition, because signals from other neurons can modulate the release of hypothalamic hormones, the hypothalamus serves as the major link between the nervous and endocrine systems. For example, the hypothalamus receives information from higher brain centers that respond to various environmental signals. Consequently, hypothalamic function is influenced by both the external and internal environments as well as by hormone feedback. Stimuli from the external environment that indirectly influence hypothalamic function include the light-dark cycle; temperature; signals from other members of the same species; and a wide variety of visual, auditory, olfactory, and sensory stimuli. The communication between other brain areas and the hypothalamus, which conveys information about the internal environment, involves electrochemical signal transmission through molecules called neurotransmitters (e.g., aspartate, dopamine, gamma-aminobutyric acid, glutamate, norepinephrine, and serotonin). The complex interplay of the actions of various neurotransmitters regulates the production and release of hormones from the hypothalamus.

The hypothalamic hormones are released into blood vessels that connect the hypothalamus and the pituitary gland (i.e., the hypothalamic-hypophyseal portal system). Because they generally promote or inhibit the release of hormones from the pituitary gland, hypothalamic hormones are commonly called releasing or inhibiting hormones. The major releasing and inhibiting hormones include the following (also see table, p. 156):

- Corticotropin-releasing hormone (CRH), which is part of the hormone system regulating carbohydrate, protein, and fat metabolism as well as sodium and water balance in the body
- Gonadotropin-releasing hormone (GnRH), which helps control sexual and reproductive functions, including pregnancy and lactation (i.e., milk production)
- Thyrotropin-releasing hormone (TRH), which is part of the hormone system controlling the metabolic processes of all cells and which contributes to the hormonal regulation of lactation
- Growth hormone-releasing hormone (GHRH), which is an essential component of the system promoting the organism’s growth
- Somatostatin, which also affects bone and muscle growth but has the opposite effect as that of GHRH
- Dopamine, a substance that functions primarily as a neurotransmitter but also has some hormonal effects, such as repressing lactation until it is needed after childbirth.

## The Pituitary and Its Major Hormones

The pituitary (also sometimes called the hypophysis) is a gland about the size of a small marble and is located in the brain directly below the hypothalamus. The pituitary gland consists of two parts: the anterior pituitary and the posterior pituitary.

### The Anterior Pituitary

The anterior pituitary produces several important hormones that either stimulate target glands (e.g., the adrenal glands, gonads, or thyroid gland) to produce target gland hormones or directly affect target organs. The pituitary hormones include adrenocorticotropic hormone (ACTH); gonadotropins; thyroid-stimulating hormone (TSH), also called thyrotropin; growth hormone (GH); and prolactin.

The first three of those hormones—ACTH, gonadotropins, and TSH—act on other glands. Thus, ACTH stimulates the adrenal cortex to produce corticosteroid hormones—primarily cortisol—as well as small amounts of female and male sex hormones. The gonadotropins comprise two molecules, luteinizing hormone (LH) and follicle-stimulating hormone (FSH). These two hormones regulate the production of female and male sex hormones in the ovaries and testes as well as the production of the germ cells—that is, the egg cells (i.e., ova) and sperm cells (i.e., spermatozoa). TSH stimulates the thyroid gland to produce and release thyroid hormone. The remaining two pituitary hormones, GH and prolactin, directly affect their target organs.

#### Growth Hormone

GH is the most abundant of the pituitary hormones. As the name implies, it plays a pivotal role in controlling the body’s growth and development. For example, it stimulates the linear growth of the bones; promotes the growth of internal organs, fat (i.e., adipose) tissue, connective tissue, endocrine glands, and muscle; and controls the development of the reproductive organs. Accordingly, the GH levels in the blood are highest during early childhood and puberty and decline thereafter. Nevertheless, even relatively low GH levels still may be important later in life, and GH deficiency may contribute to some symptoms of aging.

In addition to its growth-promoting role, GH affects carbohydrate, protein, and fat (i.e., lipid) metabolism. Thus, GH increases the levels of the sugar glucose in the blood by reducing glucose uptake by muscle cells and adipose tissue and by promoting glucose production (i.e., gluconeogenesis) from precursor molecules in the liver. (These actions are opposite to those of the hormone insulin, which is discussed in the section “The Pancreas and Its Hormones,” p. 160.) GH also enhances the uptake of amino acids from the blood into cells, as well as their incorporation into proteins, and stimulates the breakdown of lipids in adipose tissue.

To elicit these various effects, GH modulates the activities of numerous target organs, including the liver, kidneys, bone, cartilage, skeletal muscle, and adipose cells. For some of these effects, GH acts directly on the target cells. In other cases, however, GH acts indirectly by stimulating the production of a molecule called insulin-like growth factor 1 (IGF-1) in the liver and kidneys. The blood then transports IGF-1 to the target organs, where it binds to specific receptors on the cells. This interaction then may lead to the increased DNA production and cell division that underlie the growth process.

Two hypothalamic hormones control GH release: (1) GHRH, which stimulates GH release, and (2) somatostatin, which inhibits GH release. This regulatory mechanism also involves a short-loop feedback component, by which GH acts on the hypothalamus to stimulate somatostatin release. In addition, GH release is enhanced by stress, such as low blood sugar levels (i.e., hypoglycemia) or severe exercise, and by the onset of deep sleep.

Acute and chronic alcohol consumption have been shown to reduce the levels of GH and IGF-1 in the blood. Both effects have been observed in animals as well as in humans. Acute alcohol administration also reduces GH secretion in response to other stimuli that normally enhance the hormone’s release. Those deleterious effects of alcohol may be particularly harmful to adolescents, who require GH for normal development and puberty. (For more information on alcohol’s effects on puberty and growth, see the article by Dees and colleagues, pp. 165–169.)

#### Prolactin

Together with other hormones, prolactin plays a central role in the development of the female breast and in the initiation and maintenance of lactation after childbirth. Prolactin’s function in men, however, is not well understood, although excessive prolactin release can lead to reduced sex drive (i.e., libido) and impotence. Several factors control prolactin release from the anterior pituitary. For example, prolactin is released in increasing amounts in response to the rise in estrogen levels in the blood that occurs during pregnancy. In nursing women, prolactin is released in response to suckling by the infant. Several releasing and inhibitory factors from the hypothalamus also control prolactin release. The most important of those factors is dopamine, which has an inhibitory effect.

Alcohol consumption by nursing women can influence lactation both through its effects on the release of prolactin and oxytocin (see the following section) and through its effects on the milk-producing (i.e., mammary) glands and the composition of the milk. (For more information on alcohol’s effects on lactation, see the article by Heil and Subramanian, pp. 178–184.)

### The Posterior Pituitary

The posterior pituitary does not produce its own hormones; instead, it stores two hormones—vasopressin and oxytocin—that are produced by neurons in the hypothalamus. Both hormones collect at the ends of the neurons, which are located in the hypothalamus and extend to the posterior pituitary.

Vasopressin, also called arginine vasopressin (AVP), plays an important role in the body’s water and electrolyte economy. Thus, AVP release promotes the reabsorption of water from the urine in the kidneys. Through this mechanism, the body reduces urine volume and conserves water. AVP release from the pituitary is controlled by the concentration of sodium in the blood as well as by blood volume and blood pressure. For example, high blood pressure or increased blood volume results in the inhibition of AVP release. Consequently, more water is released with the urine, and both blood pressure and blood volume are reduced. Alcohol also has been shown to inhibit AVP release. Conversely, certain other drugs (e.g., nicotine and morphine) increase AVP release, as do severe pain, fear, nausea, and general anesthesia, thereby resulting in lower urine production and water retention.

Oxytocin, the second hormone stored in the posterior pituitary, stimulates the contractions of the uterus during childbirth. In nursing women, the hormone activates milk ejection in response to suckling by the infant (i.e., the so-called let-down reflex).

## The Adrenal Glands and Their Hormones

The adrenal glands are small structures located on top of the kidneys. Structurally, they consist of an outer layer (i.e., the cortex) and an inner layer (i.e., the medulla). The adrenal cortex produces numerous hormones, primarily corticosteroids (i.e., glucocorticoids and mineralocorticoids). The cortex is also the source of small amounts of sex hormones; those amounts, however, are insignificant compared with the amounts normally produced by the ovaries and testes. The adrenal medulla generates two substances—adrenaline and noradrenaline—that are released as part of the fight-or-flight response to various stress factors.

The primary glucocorticoid in humans is cortisol (also called hydro-cortisone), which helps control carbohydrate, protein, and lipid metabolism. For example, cortisol increases glucose levels in the blood by stimulating gluconeogenesis in the liver and promotes the formation of glycogen (i.e., a molecule that serves as the storage form of glucose) in the liver. Cortisol also reduces glucose uptake into muscle and adipose tissue, thereby opposing the effects of insulin. Furthermore, in various tissues, cortisol promotes protein and lipid breakdown into products (i.e., amino acids and glycerol, respectively) that can be used for gluconeogenesis.

In addition to those metabolic activities, cortisol appears to protect the body against the deleterious effects of various stress factors, including acute trauma, major surgery, severe infections, pain, blood loss, hypoglycemia, and emotional stress. All of these stress factors lead to drastic increases in the cortisol levels in the blood. For people in whom cortisol levels cannot increase (e.g., because they had their adrenal glands removed), even mild stress can be fatal. Finally, high doses of cortisol and other corticosteroids can be used medically to suppress tissue inflammation in response to injuries and to reduce the immune response to foreign molecules.

The primary mineralocorticoid in humans is aldosterone, which also helps regulate the body’s water and electrolyte balance. Its principal functions are to conserve sodium and to excrete potassium from the body. For example, aldosterone promotes the reabsorption of sodium in the kidney, thereby reducing water excretion and increasing blood volume. Similarly, aldosterone decreases the ratio of sodium to potassium concentrations in sweat and saliva, thereby preventing sodium loss via those routes. The effect can be highly beneficial in hot climates, where much sweating occurs.

In contrast to the glucocorticoids, pituitary, or hypothalamic, hormones do not regulate aldosterone release. Instead, it is controlled primarily by another hormone system, the reninangiotensin system, which also controls kidney function. In addition, the levels of sodium and potassium in the blood influence aldosterone levels.

## The Gonads and Their Hormones

The gonads (i.e., the ovaries and testes) serve two major functions. First, they produce the germ cells (i.e., ova in the ovaries and spermatozoa in the testes). Second, the gonads synthesize steroid sex hormones that are necessary for the development and function of both female and male reproductive organs and secondary sex characteristics (e.g., the adult distribution of body hair, such as facial hair in men) as well as for pregnancy, childbirth, and lactation. Three types of sex hormones exist; each with different functions: (1) estrogens (e.g., estradiol), which exert feminizing effects; (2) progestogens (e.g., progesterone), which affect the uterus in preparation for and during pregnancy; and (3) androgens (e.g., testosterone), which exert masculinizing effects. In addition to the reproductive functions, sex hormones play numerous essential roles throughout the body. For example, they affect the metabolism of carbohydrates and lipids, the cardiovascular system, and bone growth and development.

### Estrogens

The major estrogen is estradiol, which, in addition to small amounts of estrone and estriol, is produced primarily in the ovaries. Other production sites of estrogens include the corpus luteum,^2^ the placenta, and the adrenal glands. In men and postmenopausal women, most estrogens present in the circulation are derived from the conversion of testicular, adrenal, and ovarian androgens. The conversion occurs in peripheral tissues, primarily adipose tissue and skin.

The main role of estrogens is to coordinate the normal development and functioning of the female genitalia and breasts. During puberty, estrogens promote the growth of the uterus, breasts, and vagina; determine the pattern of fat deposition and distribution in the body that results in the typical female shape; regulate the pubertal growth spurt and cessation of growth at adult height; and control the development of secondary sexual characteristics. In adult women, the primary functions of estrogens include regulating the menstrual cycle, contributing to the hormonal regulation of pregnancy and lactation, and maintaining female libido. (For more information on the menstrual cycle and alcohol’s effects on it, see the article by Dees and colleagues, pp. 165–169. For more information on alcohol’s effects on the developing fetus, see the article by Gabriel and colleagues, pp. 170–177.)

During menopause, estrogen production in the ovaries ceases. The resulting reduction in estrogen levels leads to symptoms such as hot flashes, sweating, pounding of the heart (i.e., palpitations), increased irritability, anxiety, depression, and brittle bones (i.e., osteoporosis). The administration of estrogens (i.e., hormone replacement therapy) can alleviate those symptoms and reduce the risk of osteoporosis and coronary heart disease in postmenopausal women. At the same time, however, hormone replacement therapy may increase the risk of certain types of cancer (e.g., breast cancer and uterine [i.e., endometrial] cancer). Alcohol consumption has been shown to increase estrogen levels in the blood and urine, even in premenopausal women who drink two drinks or less per day (Reichman et al. 1993) and in postmenopausal women who drink less than one drink per day (Gavaler and Van Thiel 1992). These findings suggest that moderate alcohol consumption may help prevent osteoporosis and coronary heart disease in postmenopausal women. Other studies, however, have detected no consistent association between alchol consumption and increased estrogen levels (Dorgan et al. 1994; Purohit 1998). (For more information on the effects of alcohol on postmenopausal women, see the articles by Longnecker and Tseng, pp. 185–189, and Gavaler, pp. 220–227.)

### Progestogens

The ovaries produce progestogens during a certain phase of the menstrual cycle and in the placenta for most of pregnancy. Progestogens cause changes in the uterine lining in preparation for pregnancy and—together with estrogens—stimulate the development of the mammary glands in the breasts in preparation for lactation. The primary progestogen is progesterone.

### Androgens

The principal androgenic steroid is testosterone, which is secreted primarily from the testes but also, in small amounts, from the adrenal glands (both in men and women) and from the ovaries. Its main function is to stimulate the development and growth of the male genital tract. In addition, testosterone has strong protein anabolic activities—that is, it promotes protein generation, which leads to increased muscle mass. The specific functions of testosterone vary during different developmental stages, as follows:

- In the fetus, testosterone primarily ensures the development of the internal and external male genitalia
- During puberty, testosterone promotes the growth of the male sex organs and is responsible for other male developmental characteristics, such as the pubertal growth spurt and eventual cessation of growth at adult height; deepening of the voice; growth of facial, pubic, axillary, and body hair; and increase in muscularity and strength
- In the adult male, testosterone primarily serves to maintain masculinity, libido, and sexual potency as well as regulate sperm production. Testosterone levels decline slightly with age, although the drop is not as drastic as the reduction in estrogen levels in women during menopause. (For information on alcohol’s effects on male reproduction, see the article by Emanuele and Emanuele, pp.195–201.)

## The Thyroid and Its Hormones

The thyroid gland, which consists of two lobes, is located in front of the windpipe (i.e., trachea), just below the voice box (i.e., larynx). The gland produces two structurally related hormones, thyroxine (T_4_) and triiodothyronine (T_3_), that are iodinated derivatives of the amino acid tyrosine. Both hormones are collectively referred to as “thyroid hormone.” T_4_ constitutes approximately 90 percent of the hormone produced in the thyroid gland. However, T_3_ is a much more active hormone, and most of the T_4_ produced by the thyroid is converted into T_3_ in the liver and kidneys.

Thyroid hormone in general serves to increase the metabolism of almost all body tissues. For example, thyroid hormone stimulates the production of certain proteins involved in heat generation in the body, a function that is essential for maintaining body temperature in cold climates. Moreover, thyroid hormone promotes several other metabolic processes involving carbohydrates, proteins, and lipids that help generate the energy required for the body’s functions. In addition to those metabolic effects, thyroid hormone plays an essential role in the development of the central nervous system during late fetal and early postnatal developmental stages. Furthermore, thyroid hormone exerts an effect similar to that of GH on normal bone growth and maturation. Finally, thyroid hormone is required for the normal development of teeth, skin, and hair follicles as well as for the functioning of the nervous, cardiovascular, and gastrointestinal systems.

In addition to thyroid hormone, certain cells (i.e., parafollicular C cells) in the thyroid gland produce calcitonin, a hormone that helps maintain normal calcium levels in the blood. Specifically, calcitonin lowers calcium levels in the blood by reducing the release of calcium from the bones; inhibiting the constant erosion of bones (i.e., bone resorption), which also releases calcium; and inhibiting the reabsorption of calcium in the kidneys. Those effects are opposite to those of parathyroid hormone (PTH), which is discussed in the following section.

## The Parathyroid Glands and Their Hormones

The parathyroid glands are four pea-sized bodies located behind the thyroid gland that produce PTH. This hormone increases calcium levels in the blood, helping to maintain bone quality and an adequate supply of calcium, which is needed for numerous functions throughout the body (e.g., muscle movement and signal transmission within cells). Specifically, PTH causes reabsorption of calcium from and excretion of phosphate in the urine. PTH also promotes the release of stored calcium from the bones as well as bone resorption, both of which increase calcium levels in the blood. Finally, PTH stimulates the absorption of calcium from the food in the gastrointestinal tract. Consistent with PTH’s central role in calcium metabolism, the release of this hormone is not controlled by pituitary hormones but by the calcium levels in the blood. Thus, low calcium levels stimulate PTH release, whereas high calcium levels suppress it.

Many of the functions of PTH require or are facilitated by a substance called 1,25-dihydroxycholecalciferol, a derivative of vitamin D. In addition, numerous other hormones are involved in regulating the body’s calcium levels and bone metabolism, including estrogens, glucocorticoids, and growth hormone. (For more information on the hormonal control of bone and calcium metabolism and on alcohol’s effects on those systems, see the article by Sampson, pp. 190–194.)

## The Pancreas and Its Hormones

The pancreas is located in the abdomen, behind the stomach, and serves two distinctly different functions. First, it acts as an exocrine organ, because the majority of pancreatic cells produce various digestive enzymes that are secreted into the gut and which are essential for the effective digestion of food. Second, the pancreas serves as an endocrine organ, because certain cell clusters (i.e., the Islets of Langerhans) produce two hormones—insulin and glucagon—that are released into the blood and play pivotal roles in blood glucose regulation.

### Insulin

Insulin is produced in the beta cells of the Islets of Langerhans. Its primary purpose is to lower blood glucose levels; in fact, insulin is the only blood sugar-lowering hormone in the body. To this end, insulin promotes the formation of storage forms of energy (e.g., glycogen, proteins, and lipids) and suppresses the breakdown of those stored nutrients. Accordingly, the target organs of insulin are primarily those that are specialized for energy storage, such as the liver, muscles, and adipose tissue. Specifically, insulin has the following metabolic effects:

- Promotes glucose uptake into cells and its conversion into glycogen, stimulates the breakdown of glucose, and inhibits gluconeogenesis
- Stimulates the transport of amino acids into cells and protein synthesis in muscle cells, thereby lowering the levels of amino acids available for gluconeogenesis in the liver
- Increases fat synthesis in the liver and adipose tissue, thereby lowering the levels of glycerol, which also can serve as a starting material for gluconeogenesis.

The release of insulin is controlled by various factors, including blood glucose levels; other islet hormones (e.g., glucagon); and, indirectly, other hormones that alter blood glucose levels (e.g., GH, glucocorticoids, and thyroid hormone).

### Glucagon

The second blood-sugar–regulating pancreatic hormone is glucagon, which is produced in the alpha cells of the Islets of Langerhans. Glucagon increases blood glucose levels; accordingly, its main actions generally are opposite to those of insulin. For example, glucagon increases glycogen breakdown and gluconeogenesis in the liver as well as the breakdown of lipids and proteins. The release of glucagon is regulated by many of the same factors as is insulin’s release, but sometimes with the opposite effect. Thus, an increase in blood glucose levels stimulates insulin release but inhibits glucagon release.

A finely tuned balance between the activities of insulin and glucagon is essential for maintaining blood sugar levels. Accordingly, disturbances of that balance, such as an insulin deficiency or an inability of the body to respond adequately to insulin, result in serious disorders, such as diabetes mellitus. (For more information on diabetes and on alcohol’s effects on insulin, glucagon, and the management of diabetes, see the article by Emanuele and colleagues, pp. 211–219.)

## Hormone Systems

**Figure 3:**
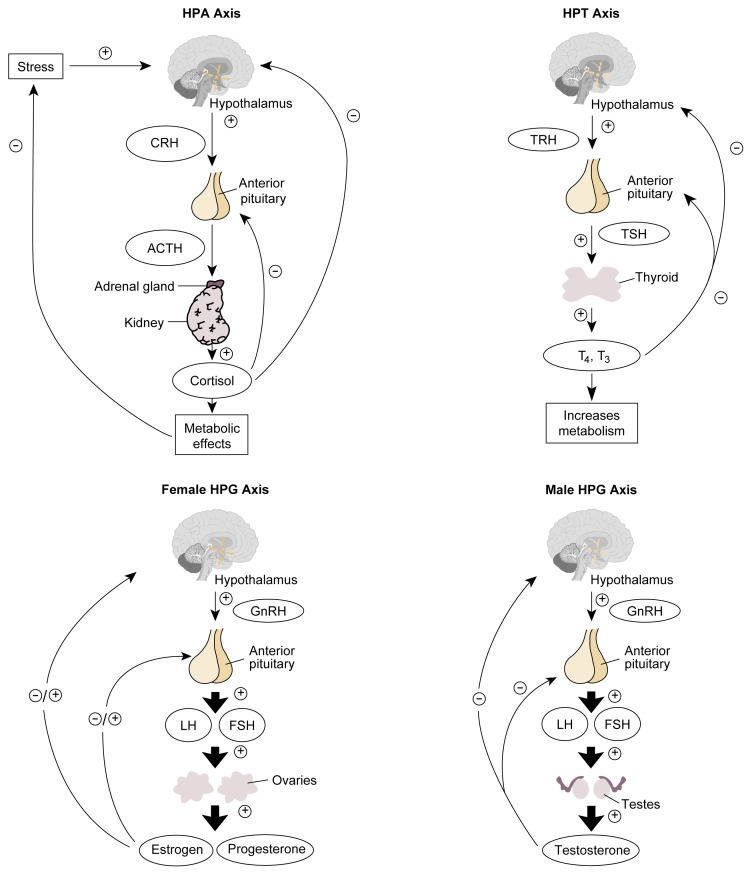
Schematic representation of the HPA, HPG, and HPT axes. For each system, the hypothalamus secretes releasing hormones (i.e., CRH, GnRH, and TRH) that act on the pituitary gland. In response to those stimuli, the pituitary gland releases ACTH, gonadotropins (i.e., LH and FSH), or TSH. ACTH activates the adrenal glands to release cortisol, which induces metabolic effects. Cortisol also acts back on the hypothalamus and pituitary gland by negative feedback. LH and FSH in women stimulate the ovaries to produce estrogens and progesterone. Depending on the phase of the menstrual cycle, those hormones act back on the hypothalamus and pituitary gland in either a stimulatory or inhibitory manner. In men, LH stimulates the testes to release testosterone, which feeds back on the hypothalamus and pituitary. Finally, TSH stimulates the thyroid gland to produce the thyroid hormones T_3_ and T_4_, both of which increase cell metabolism as well as feed back on the hypothalamus and pituitary. NOTE: = stimulates; = inhibits; ACTH = adrenocorticotropic hormone; CRH = corticotropin-releasing hormone; FSH = follicle-stimulating hormone; GnRH = gonadotropin-releasing hormone; HPA = hypothalamic-pituitary-adrenal; HPG = hypothalamic-pituitary-gonadal; HPT = hypothalamic-pituitary-thyroid; LH = luteinizing hormone; T_3_ = triiodothyronine; T_4_ = thyroxine; TRH = thyrotropin-releasing hormone; TSH = thyroid-stimulating hormone.

As this article has indicated in describing the various endocrine glands and their hormones, some hormones are controlled directly by the metabolic pathways that they influence. For example, blood sugar levels directly control insulin and glucagon release by the pancreas, and calcium levels in the blood regulate PTH release. Conversely, many hormones produced by target glands are regulated by pituitary hormones, which in turn are controlled by hypothalamic hormones. Examples of such regulatory hormonal cascades include the hypothalamic-pituitary-adrenal (HPA) axis, the hypothalamic-pituitary-gonadal (HPG) axis, and the hypothalamic-pituitary-thyroidal (HPT) axis, which are described briefly in the following sections (see figure 3, p.162).

### The HPA Axis

Activation of the HPA axis, which regulates various metabolic functions, is initiated with the release of CRH from the hypothalamus. This release occurs in response to various stimuli, including almost any type of physical or psychological stress; during the normal sleep-wake cycle; and in response to certain neurotransmitters. CRH then stimulates the anterior pituitary to produce ACTH. (In addition to CRH, AVP from the hypothalamus also can stimulate ACTH release). ACTH, in turn, activates adrenal hormone production, primarily of cortisol, which mediates the specific physiological effects of this hormone system.

The activity of the HPA axis is regulated by negative feedback mechanisms. Thus, increased cortisol levels repress CRH release by the hypothalamus and ACTH release by the pituitary. In addition, ACTH can directly inhibit hypothalamic CRH release.

Any disturbances in the HPA axis can result in serious medical consequences. For example, insufficient hormone production by the adrenal cortex causes Addison’s disease, which is characterized by muscle weakness, dehydration, loss of appetite (i.e., anorexia), nausea, vomiting, diarrhea, fever, abdominal pain, tiredness, and malaise. Patients with this disease exhibit low levels of plasma cortisol but high levels of ACTH. The increase in ACTH levels represents a vain attempt by the pituitary to stimulate hormone production in the unresponsive adrenal cortex.

Equally deleterious is the excessive glucocorticoid production that results from excess ACTH release (i.e., Cushing’s syndrome). Those patients experience symptoms such as muscle weakness and wasting, back pain from osteoporosis, a tendency to bruise easily, redistribution of body fat (i.e., a rounded “moon” face, prominent abdomen, and thin legs), and various psychological disturbances. Because of the negative feedback mechanism of the HPA axis, the patient’s cortisol levels are high and the ACTH levels are low.

Both acute and chronic alcohol consumption have been shown to activate the HPA axis, and some drinkers develop a so-called pseudo-Cushing’s syndrome that disappears with abstinence (Veldman and Meinders 1996; Emanuele and Emanuele 1997). (For more information on alcohol’s effect on the HPA axis and its relation to alcohol craving, see the article by Gianoulakis, pp. 202–210.)

### The HPG Axis

In both men and women, the HPG axis is the hormone system that controls the release of sex hormones. In both genders, the system is activated by GnRH, which is released regularly in short bursts from the hypothalamus. GnRH then stimulates the release of FSH and LH from the anterior pituitary.

In men, LH stimulates certain cells in the testes (i.e., Leydig cells) to release testosterone. FSH and testosterone are key regulators of another set of testicular cells (i.e., Sertoli cells), which support and nourish the sperm cells during their maturation. The HPG axis in men is regulated through a variety of factors. For example, testosterone is part of a negative feedback mechanism that inhibits GnRH release by the hypothalamus and LH release by the pituitary. In addition, the Sertoli cells secrete a substance called inhibin, which prevents FSH release from the pituitary. Finally, the Leydig cells and, to a lesser extent, the Sertoli cells produce a substance called activin, which stimulates FSH secretion and thus has the opposite effects of inhibin.

In women, during the menstrual cycle, LH and FSH stimulate the ovarian follicle that contains the maturing egg to produce estradiol. After ovulation has occurred, LH also promotes production of progesterone and estradiol by the corpus luteum. Both hormones participate in a negative feedback mechanism through most of the menstrual cycle, suppressing GnRH release from the hypothalamus and LH release from the pituitary. Shortly before ovulation, however, a positive feedback mechanism is activated by which estradiol actually enhances LH release from the pituitary. The resulting surge in LH levels ultimately leads to ovulation, the formation of the corpus luteum, and progesterone release. Progesterone exerts a negative feedback on LH and FSH release, causing LH levels to decline again. In addition to those mechanisms, FSH release from the pituitary is regulated by inhibin, a substance produced by certain cells in the ovarian follicle.

Both acute and chronic alcohol consumption can interfere with the normal functioning of the HPG axis, resulting in reduced fertility or even infertility in both men and women and in menstrual disturbances in women. (For more information on alcohol’s effects on the HPG axis in women and men, see the articles by Dees and colleagues, pp. 165–169, and by Emanuele and Emanuele, pp. 195–201.)

### The HPT Axis

The hormones that make up the HPT axis control the metabolic processes of all cells in the body and are therefore crucial for the organism to function normally. The secretion of TRH from the hypothalamus activates the HPT axis. After reaching the pituitary, TRH stimulates the release of TSH, which in turn promotes the production and release of T_4_ and T_3_ by the thyroid gland. Negative feed-back effects of T_4_ and T_3_ on both the hypothalamus and the pituitary regulate the HPT system. (For a summary of alcohol’s effects on the HPT axis, see sidebar, p. 163.)

**Alcohol’s Effects on the Hypothalamic-Pituitary-Thyroid Axis**

One of the essential hormonal systems regulating normal body functioning is the hypothalamic-pituitary-thyroid (HPT) axis, which controls the metabolism of all cells. As with other hormone systems, alcohol consumption under certain conditions can modify the release of hormones involved in this axis. In healthy nonalcoholics, alcohol consumption does not appear to induce any significant changes in the HPT axis (Emanuele and Emanuele 1997). Conversely, some effects of alcohol on the HPT axis have been observed in alcoholics. The effects differ depending on the drinking status of the alcoholics studied. In alcoholics undergoing withdrawal, baseline levels of thyroid hormone (i.e., T_3_ and T_4_) in the blood differ only minimally from those in nonalcoholics. The ability of hypothalamic thyrotropin-releasing hormone (TRH) to activate the release of thyroid-stimulating hormone (TSH) from the pituitary, however, is impaired in these alcoholics (Emanuele and Emanuele 1997). This “blunting” effect may result from alcohol’s influence on the neurotransmitter dopamine. Dopamine produced in the hypothalamus acts not only as a neurotransmitter but also as a hormone in that it inhibits the release of both TSH and prolactin from the pituitary. Alcohol has been shown to increase dopaminergic activity and thereby may suppress the TSH response to TRH. This hypothesis is supported by the fact that prolactin release in response to TRH also is blunted in alcoholics undergoing withdrawal.

Alcohol’s effects on the HPT axis are even more complex in abstinent alcoholics (Garbutt et al. 1995). In those people, the baseline levels of T_3_ and sometimes T_4_ are lower than in nonalcoholics. It is unclear, however, if this change represents a direct effect of long-term alcohol consumption or results from co-occurring alcohol-related illnesses, because thyroid hormone levels are often reduced in patients with acute or chronic non-thyroid–related illnesses, such as sepsis, burns, or major trauma. In addition to the reduced thyroid hormone levels, however, the TSH response to TRH remains blunted in abstinent alcoholics, whereas the prolactin response to TRH has returned to normal levels. This observation indicates that a factor other than dopamine likely contributes to this effect, although the exact mechanisms are unknown.

Finally, some intriguing findings have suggested that abnormal responses of the HPT axis may represent a marker for a person’s vulnerability to alcoholism. Thus, some people who are at high risk for developing alcoholism, such as nonalcoholic sons of alcoholic fathers, tend to exhibit a blunted TSH response to TRH (Emanuele and Emanuele 1997). These observations still require further investigation, however, for researchers to fully understand their significance.

***—Susanne Hiller-Sturmhöfel and Andrzej Bartke***

ReferencesEmanueleNEmanueleMAThe endocrine system: Alcohol alters critical hormonal balanceAlcohol Health & Research World2115364199715706763PMC6826794GarbuttJCSilvaSGMasonGAThyrotropin-releasing hormone (TRH): Clinical neuroendocrine and neurobehavioral findings of relevance to alcoholismWatsonRRDrug and Alcohol Abuse Reviews: Volume 6. Alcohol and HormonesTotowa, NJHumana Press1995127145

## Summary

The neuroendocrine system is a highly complex and tightly controlled network of hormones released by endocrine glands throughout the body. The levels of some of the hormones are regulated in a fairly straightforward manner by the end products that they influence. Thus, blood sugar levels primarily regulate insulin and glucagon release by the pancreas. Other hormones (e.g., those of the HPA, HPG, and HPT axes) are parts of hormone cascades whose activities are controlled through elaborate feedback mechanisms. In addition, numerous indirect interactions exist between the various hormone systems governing body functioning. For example, hormones such as GH and thyroid hormone, through their effects on cellular metabolism, may modify blood sugar levels and, accordingly, insulin release. Similarly, alcohol’s effects on one hormone system may have indirect consequences for other systems, thereby contributing to alcohol’s influences on the functioning of virtually every organ in the body. It is important to keep this interconnectedness of neuroendocrine systems in mind when analyzing alcohol’s impact on various hormones, which are described in the remaining articles in this issue.

## Footnotes

1 Conversely, exocrine glands (e.g., sweat glands and salivary glands) release their secretions to the outside of the body (e.g., sweat) or into a hollow space that is open to the outside (e.g., saliva released into the mouth).

2 The corpus luteum is a group of cells derived from the follicle that releases the ovum during a particular menstrual cycle.
